# Approach to multifunctional device platform with epitaxial graphene on transition metal oxide

**DOI:** 10.1038/srep14374

**Published:** 2015-09-23

**Authors:** Jeongho Park, Tyson Back, William C. Mitchel, Steve S. Kim, Said Elhamri, John Boeckl, Steven B. Fairchild, Rajesh Naik, Andrey A. Voevodin

**Affiliations:** 1 Air Force Research Laboratory, Materials and Manufacturing Directorate (AFRL/RXA) Wright-Patterson AFB, OH 45433-7707; 2 University of Dayton Research Institute, Dayton, Ohio 45469-0170, USA; 3 Center of Excellence for Thin Film Research and Surface Engineering, University of Dayton, Dayton, Ohio 45469-0170, USA; 4 Departments of Physics, University of Dayton, Dayton, Ohio 45469.

## Abstract

Heterostructures consisting of two-dimensional materials have shown new physical phenomena, novel electronic and optical properties, and new device concepts not observed in bulk material systems or purely three dimensional heterostructures. These new effects originated mostly from the van der Waals interaction between the different layers. Here we report that a new optical and electronic device platform can be provided by heterostructures of 2D graphene with a metal oxide (TiO_2_). Our novel direct synthesis of graphene/TiO_2_ heterostructure is achieved by C_60_ deposition on transition Ti metal surface using a molecular beam epitaxy approach and O_2_ intercalation method, which is compatible with wafer scale growth of heterostructures. As-grown heterostructures exhibit inherent photosensitivity in the visible light spectrum with high photo responsivity. The photo sensitivity is 25 times higher than that of reported graphene photo detectors. The improved responsivity is attributed to optical transitions between O 2p orbitals in the valence band of TiO_2_ and C 2p orbitals in the conduction band of graphene enabled by Coulomb interactions at the interface. In addition, this heterostructure provides a platform for realization of bottom gated graphene field effect devices with graphene and TiO_2_ playing the roles of channel and gate dielectric layers, respectively.

Graphene (Gr) is a very attractive material for diverse optoelectronic and electronic device applications due to its extraordinary transport properties, broad spectral-bandwidth, and fast response time^1^,^2^,^3^,^4^. For many applications, dielectrics layers such as SiO_2_ play a crucial role in device operation. In graphene field effect transistors (GFET), graphene provides the active layer where the channel carriers are modulated by gate biasing through the dielectric layer. In graphene optical devices a dielectric layer is needed to induce a built-in potential within the structure so that photo-excited electrons can be extracted from the photosensitive materials into the graphene layer on top. Currently the photosensitive 2D materials such as MoS_2_ or WS_2_ are widely investigated to enhance photo-responsibility. Recent studies of graphene/photosensitive 2D heterostructures have revealed new physical phenomena such as Moire patterns^5^,^6^, new Dirac points^7^, and the Hofstadter butterfly^8^,^9^. These originate from the van der Waals interaction at the interface. The observation of significant improvement of quantum efficiency in Gr/WS_2_/Gr heterostructure resulted from Van Hove singularities^10^ originated from van der Waals interaction. Due to the lack of direct growth methods, however, fabrication is typically done by exfoliation or transfer methods. Here, we propose new direct growth approach to structure graphene/a photosensitive material and report its dual function (bottom gated FET and photodetector) that make it a unique hybrid device. In this studies, TiO_2_ has been considered since it is a well-known material that has been widely used for light harvesting^11^ as well as a high dielectric constant (~100). These dual characteristics make TiO_2_ a very attractive material for the realization of both electronic and photonic devices. The schematic diagram of our growth approach is shown in Fig. 1a. The as-grown heterostructure displays both photosensitivity under visible light and field effect phenomena when the gate voltage through the underneath TiO_2_ dielectric layer. We demonstrate this dual functionality by depositing two top metal contacts on the Gr/TiO_2_ heterostructure to serve as source and drain electrodes. The gating voltage is applied through the underlying TiO_2_ on n-type SiC in a bottom gated FET configuration. With the same device configuration, the photosensitivity under visible light illumination is measured by the change of photoconductivity with zero gate voltage. We believe that the observed photo response at zero gate voltage is caused by the optical transitions between the valence band maximum, associated O 2p orbitals in TiO_2_, and the conduction band minima, formed by the C 2p orbitals of the graphene in the Gr/TiO_2_ heterostructure. The schematic diagrams of device and measurement configurations are shown in Fig. 1b,c. The electrical transport experiments to characterize Gr/TiO_2_ heterostructure have been conducted in air.

## Results

### The growth of graphene

To investigate the graphene growth on Ti metal surfaces, the growth time and C_60_ flux dependence were studied. The growth temperature was kept at 1400 °C during these experiments. First, the C_60_ flux was held at 1.6 × 10^−7^ Torr while the growth time varied. As growth time increased, the 2D peak position shifted linearly to lower wavenumber. After 30 min. growth, the 2D peak was located at ~2712 ± 5 cm^−1^. With longer growth time (90min.), it shifted to 2705 ± 1.5 cm^−1^. The FWHM of the 2D peak showed similar behavior to peak position. The value of the FHWM decreased from 71 ± 9 cm^−1^ (30 min. growth) to 56.5 ± 2 cm^−1^ (90 min. growth). The quality of the graphene was estimated from the ratio of the D and G peaks. The growth time dependence of the D/G ratio also exhibited similar behavior to position and FWHM of the 2D peak. Increased growth time resulted in a decrease of the D/G ratio from 0.32 ± 0.07 (30 min. growth) to 0.23 ± 0.01 (90 min. growth). The C_60_ flux dependence with two different fluxes was also evaluated. The growth was done at 1400 °C for 2 hr. With increasing flux from 0.8 × 10^−7^ to 1.6 × 10^−7^ Torr, we observed improvement of graphene quality with a decrease of the D/G ratio and 2D FWHM by 40% and 20%, respectively. The peak position also shifts to lower wavenumber from 2706 cm^−1^ to 2703 cm^−1^. Next, the effect of O_2_ intercalation at 500 °C at various times was studied with Raman analysis. The 2D peak position shifted slightly to lower wavenumber. A 4hr. intercalation led a 2D peak shift of only 5 cm^−1^, to lower wavenumber. The 2D FWHM showed entirely different behavior. Initially it broadened compared to that of as-grown graphene (12 ± 5 cm^−1^ for 45 min. intercalation) and then started to narrow (13 ± 3 cm^−1^ for 2 hr. intercalation). The graphene quality variation was estimated by comparing the D/G ratio before and after intercalation. We did not observed any variation of the ratio for intercalation up to 60 min. but a significant increase was observed beyond 60 min.

### The comparison of graphene grown on Ti and TiO_2_ surface

Graphene growth was attempted on both Ti and TiO_2_ surfaces. Figure 2 shows (a) C 1s, and (b) Ti 2p regions of the X-ray photoelectron spectroscopy (XPS) spectra after C_60_ deposition on both TiO_2_ (red color curve) and Ti (blue color curve). As seen in Fig. 2a, the C 1s region exhibits two distinct peaks, located at 284.7 eV and 281.5 eV, on both surfaces. The higher and lower binding energy peaks are attributed to graphene and TiC, respectively. Typical Ti 2p_3/2_ and Ti 2p_1/2_ peaks for TiO_2_ are located at 459.1 eV and 464.8 eV respectively. However, our results (Fig. 2b) show the doublet peaks are located at 454.7 eV and 460.7 eV. The peak positions and 6.0 eV peak separation of the Ti 2p doublet agree well with those of TiC^12^. The O 1s peak for TiO_2_ is typically located at 530.6 eV but our measurement (not shown here) shows no O 1s peak corresponding to TiO_2_. Therefore, we conclude that C_60_ deposition results in graphene and TiC formation on both Ti and TiO_2_. The formation of TiC upon C_60_ deposition could be explained by a carbon thermal reduction reaction^13^. A Gibbs free energy calculation^13^ shows that TiO_2_ reacts with carbon to form TiC and releases CO gas above 1300 °C. The high substrate temperature (1400 °C) during C_60_ deposition could initiate carbon thermal reduction reactions between molecular C_60_ and the TiO_2_ surface.

### Intercalation Experiments

Intercalation between epitaxial graphene and its substrate has been widely studied as a method of decoupling the strong interaction from the substrate^14^,^15^,^16^,^17^, to modify the chemical composition of the layer under the graphene^18^, and to enable exfoliation^19^. Furthermore, TiO_2_ has been obtained from oxidation of TiC in the temperature range from 623 K to 1073 K in air^20^. Consequently, the conversion of TiC into TiO_2_ was accomplished with an O_2_ intercalation procedure. Intercalation was conducted under controlled O_2_ pressure (10^−4^ Torr) in the XPS chamber (details in Method). Figure 3 shows the evolution of the (a) C 1s, (b) Ti 2p, and (c) O 1s spectra as O_2_ intercalation progressed. Besides peaks for as-grown graphene at 284.7eV and TiC at 281.5 eV, three peaks associated with C-O (286.1 eV), C = O (288.1 eV), and the π → π* (291.5 eV) transition were observed in the C 1s spectra (not shown here). Since oxygen-carbon bonding was observed in the as-grown sample as well, the existence of C and O bonding might be due to air exposure during transfer from the growth chamber to the XPS chamber. In as-grown graphene/Ti structure, graphene peak is located at 284.7 eV with ~1.2 eV FWHM. However, the shift of peak position to 284.2 eV and the FHWM narrowing from 1.2 eV to 1 eV were observed as intercalation proceeds. We attribute the shift in binding energy to substrate interactions between the Ti and TiO_2_. The conversion of TiC into TiO_2_ during intercalation is shown in Fig. 3b. The Ti^4+^ cation doublet located at 458.8 eV and 464.6 eV and the metallic type bonding (titanium carbide) Ti^0^ peaks located at 454.7 eV and 460.9 eV were observed. These peaks are associated with Ti 2p_3/2_ and Ti 2p_1/2_ transitions, respectively. The evolution of chemical composition at different temperatures and intercalation times is also shown in Fig. 3a–c At 500 °C, the TiC C 1s peak intensity decreases as intercalation proceeds as seen in Fig. 3a (three curves on the bottom). This peak was still observable after 12 hrs. of intercalation at this temperature. A higher intercalation temperature yielded a more rapid reduction of the TiC peak intensity. At 700 °C, the TiC peak disappeared after a 2 hr. intercalation while 6 hr. intercalation was required at 600 °C. During the initial stage of intercalation, we observed two oxide states such as Ti^4+^ and Ti^3+^ along with TiC peaks (Detail in Supplementary Information). TiC related peaks in the C 1s and the Ti 2p spectra were not observed after 6 hr intercalation at 600 °C, which indicates a full conversion of TiC into TiO_2_. A further increase of temperature to 700 °C showed even faster conversion of TiC than was observed at 600 °C. The 2 hr. intercalation fully converted all of the metallic TiC layers into insulating TiO_2_ layers as shown in Fig. 3 (top two curves in each panel). The O 1s peak (Fig. 3c) for TiO_2_ starts to appear after 1 hr intercalation (not shown here) and its peak intensity increases with further intercalation. We calculated the activation energy for TiO_2_ conversion by O_2_ intercalation and graphene etching by the O_2_ (detail in Method). The calculated activation energy for TiO_2_ conversion is about 0.29 eV while activation for graphene etching is 0.41 eV, which indicate that TiO_2_ conversion is the dominant reaction in our intercalation experiments.

### Raman Analysis

The C_60_ deposition on Ti metal and O_2_ intercalation were also investigated with Raman measurements. Figure 4 exhibits Raman spectra for an as-grown heterostructure and one intercalated under O_2_ exposure. In the as-grown sample, Raman measurements show the typical Raman fingerprint of graphene with the presence of the 2D, G, and D peaks. Importantly, a fit to the 2D peak gave a single Lorentizan at ~2700 cm^−1^. Typically, C_60_ deposition on SiC resulted in Bernal stacked few layer graphene^21^ and a 2D peak best represented by multiple Lorentizans centered at ~2750 cm^−1^. This suggests that graphene on Ti metal is not Bernal stacked but is very similar to turbostractic stacking of graphene^20^. After O_2_ intercalation (Fig. 4a, red color spectrum), the 2D peak shifted to lower wavenumber and the FWHM decreased. The formation of underneath TiO_2_ layer (E_g_ = 3.2 eV) after O_2_ intercalation enhances the transmittance of visible incident Raman laser into SiC substrate. It results in Raman activation of SiC substrate and convolution of SiC and graphene Raman spectrum. The formation of TiO_2_ after intercalation was confirmed by observation of TiO_2_ vibration modes (Fig. 4b). TiO_2_ has several Raman active vibration modes at 143 cm^−1^ and 640 cm^−1^ (E_g_), an A_1g_ and B_1g_ mode doublet at 516 cm^−1^, and a B_1g_ mode at 399 cm^−1^ 22. Other than the graphene peaks, as-grown graphene only showed SiC Raman peaks such as the E_2_, A_1_, and E_1_ modes (black colored annotations), while two distinct TiO_2_ related Raman peaks (orange color annotations) were observed after O_2_ intercalation. The peaks at ~143 cm^−1^ and ~640 cm^−1^ are attributed to the E_g_ mode (O-Ti-O bending vibration) of anatase TiO_2_.

### Field Effect Transistors with Gr/TiO_2_ heterostructure

**Figure 1:**
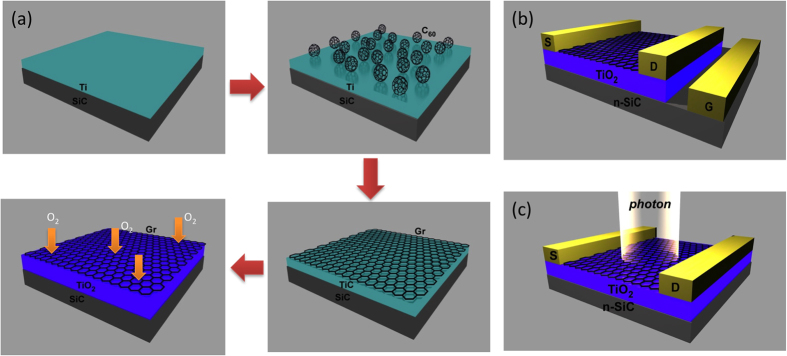
The schematic diagram of Gr/TiO_2_ structure growth and the configuration of its electronic and optical device. (**a**) The C_60_ is deposited on Ti/SiC to grow graphene. After C_60_ deposition, Ti metal converts into TiC by carbon reduction. By O_2_ intercalation of graphene/TiC/SiC structure, the finial graphene/TiO_2_/SiC heterostructure is obtained. (**b**) Configuration of bottom gated FET device. (**c**) Configuration of photo response measurement.

**Figure 2:**
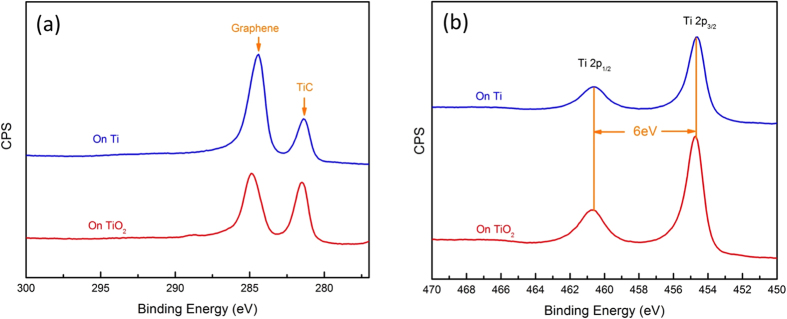
XPS spectra of graphene grown on Ti metal and TiO_2_ surface. (**a)** High resolution C 1s spectra for Gr/Ti (blue color) and Gr/TiO_2_ (red color). C 1s peaks for graphene and TiC are located at 284.7 eV and 281.5 eV on both surfaces, respectively. (**b**) Ti 2p spectra of Gr/Ti (blue color) and Gr/TiO_2_ (red color). Ti_3/2_ and Ti_1/2_ doublet lines are located at 459.1 eV and 464.8 eV, respectively.

**Figure 3:**
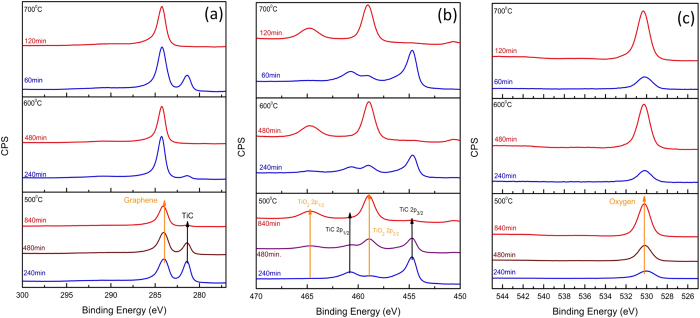
The temporal evolution of C 1s, Ti 2p, and O 1s XPS spectra during O_2_ intercalation at three different intercalation temperatures (500 °C, 600 °C, and 700 °C). Three samples (Gr/Ti/n-SiC) grown at the same growth condition were used for each temperature dependent experiment. At each intercalation temperature, the spectra were normalized to TiC C1s peak, TiC Ti 2p_2/3_, O1s peak of bottom spectrum (blue colored curve) in the each panel. (**a**) The change of carbon C1s a as function of temperature and time. (**b**) The transition from TiC to TiO_2_ during O_2_ intercalation. Ti 2p doublet of TiC diminishes and that for TiO_2_ start to appear as O_2_ intercalation proceeds. (**c**) The enhancement of O 1s peak intensity associated with TiO_2_ as O_2_ intercalation proceeds.

**Figure 4:**
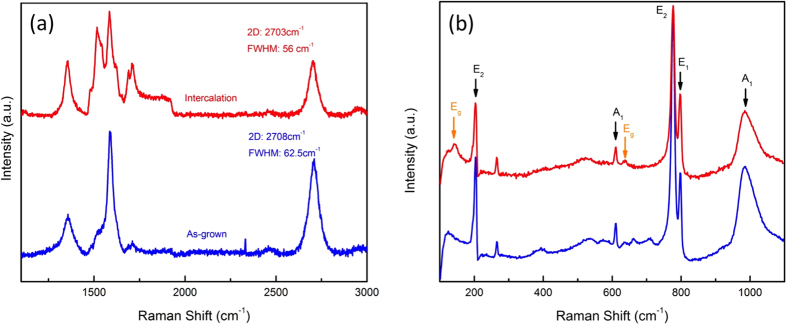
Raman characteristics of C_60_ deposition on Ti metal surface. O_2_ intercalation was performed under 1 × 10^−4^Torr oxygen partial pressure. (**a**) Comparison of Raman spectra between as-grown and O_2_ intercalated samples. Intercalated sample showed 2D peak shift to lower wavenumber and narrowing of 2D peak. (**b**) The Raman signature of TiO_2_ formation after O_2_ intercalation. The anatase TiO_2_ Raman signature (E_g_ vibration mode) was clearly observed.

**Figure 5:**
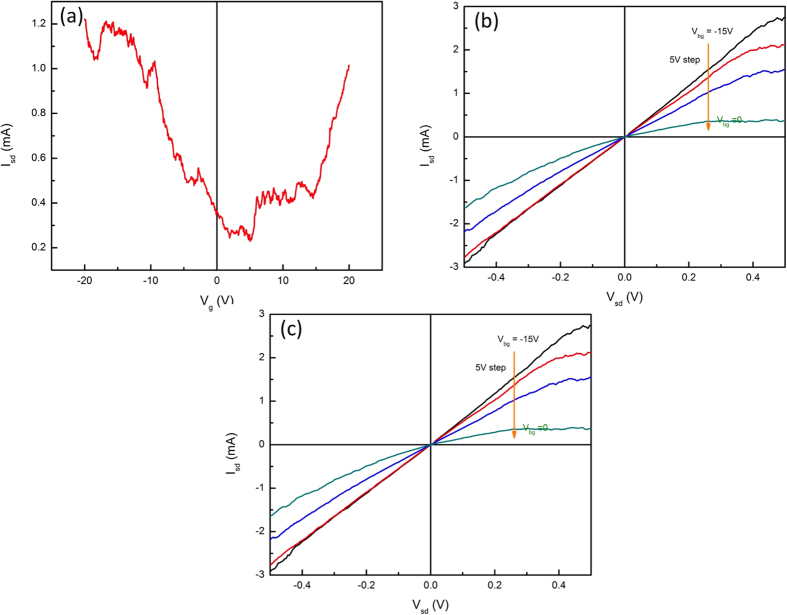
Room temperature electrical characteristics of bottom gated Gr/TiO_2_ FET device with a large area graphene channel. (**a**) Transfer characteristics (I_sd_ vs V_g_) of Gr/TiO_2_ FET at V_sd_ = 0.2 V. The charge neutral point was observed at ~4 V. (**b,c**). I_sd_ vs. V_sd_ output characteristics at negative and positive back gate voltage (V_bg_), respectively. V_bg_ was swept from −15 V to 0 V and from 5 V to 15 V with 5 V step, respectively.

To explore the electrical properties of our Gr/TiO_2_/n-SiC heterostructures we fabricated bottom gated FET devices. Ti/Au contacts were deposited for source and drain contacts on top of the 1 cm × 1 cm samples.  The channel length was about 6 mm. The schematic diagram of GFET structure is shown in Fig. 1b. First, the gate leakage current was measured to ensure isolation between the graphene and the n-SiC substrate. The measured gate leakage current was on the order of picoamps over the gate voltage range of ±10 V (See supplementary Information), indicating strong isolation was provided by the TiO_2_. The gate response was measured for a gate voltage range of ±20 V and is shown in Fig. 5a. Starting with hole-doped graphene the current flow between the source and drain decreases as the gate voltage decreased to ~3 V. Beyond this voltage, the source-drain current starts to increase as gate voltage goes negative, showing the characteristic V-shaped ambipolar behavior of graphene. The charge neutral point (CNP) occurs at V_g_ ≈ +3 V, which indicates that the intercalated graphene exhibits hole doping. Figure 5b and c show that our GFET device exhibits typical transfer characteristic of FET devices. Gate modulation experiments were performed in both hole and electron doped regimes. The drain voltage was swept from −0.5 V to 0.5 V. In the hole doped region, the gate voltage was varied from −15 V to 0 V with 5 V steps. As shown in Fig. 5b, reasonable gate modulation was demonstrated. Opposite behavior was observed in the positive gate voltage region (Fig. 5c). Here, the source-drain current increased as gate voltage increased. Note that large drain current was obtained at low drain voltage and gate voltage (I_d_ ≈ 2.9 mA at V_g_ = 15 V and V_d_ = 0.5 V). These results might be due a high capacitance due to the TiO_2_ dielectric layer.

### Photosensitivity of Gr/TiO_2_ Heterostructure

**Figure 6:**
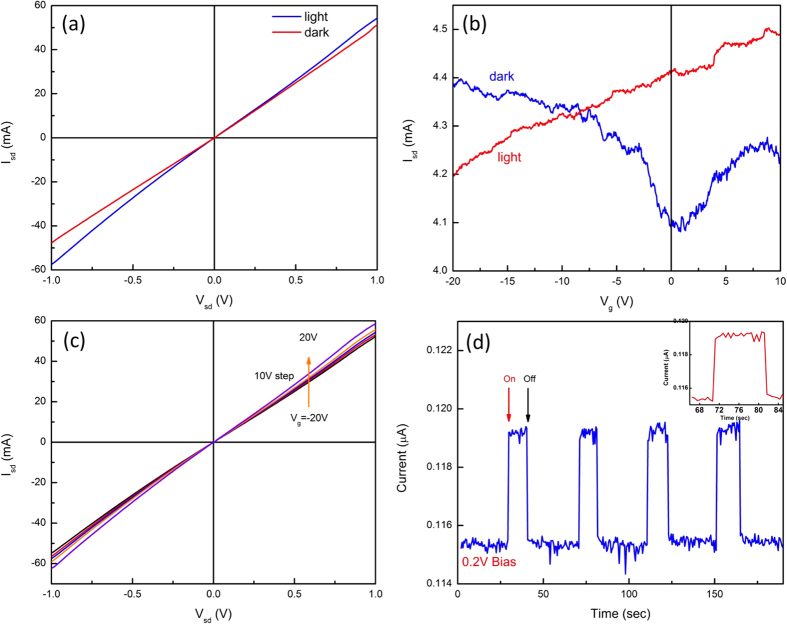
Photo-response of Gr/TiO_2_ heterostructure at zero gate voltage. (**a**) I_sd_ vs. V_sd_ output characteristics in the dark (red color plot) and under visible light illumination (blue color plot). (A) white LED (P_o_ = 36 mW) was used to illuminate the device. (**b**) The comparison of transfer characteristics (I_sd_ vs V_g_) in the dark (red color plot) and under illumination (blue color plot) at V_sd_ = 0.2 V. In the dark, the Gr/TiO_2_ heterostructure showed typical graphene ambipolar transfer characteristics. Under illumination, a linear transfer characteristic is exhibited, (**c**) Output characteristic of Gr/TiO_2_ heterostructure under illumination at different gate bias voltages from −20 V to 20 V with 5 V steps. (**d)** The on/off temporal response of Gr/TiO_2_ heterostructures at zero gate bias. V_sd_ = 0.2 V was applied during measurement. Insert: the zoomed plot showing fast photo-response behavior.

In addition to its electronic device applications as a gate dielectric material, TiO_2_ has been widely used as photosensitive material. However, its large bandgap (3.2 eV) has restricted its use to ultraviolet applications since the photo response is only activated under ultraviolet radiation. Several approaches have been proposed to extend its photo response to a broader range such as dye sensitization, doping, and coupling with semiconductors^23^,^24^,^25^. To explore possible photonic applications we measured the photosensitivity of our Gr/TiO_2_/n-SiC heterostructures at room temperature (RT). The schematic diagram of measurement is shown in Fig. 1c. Figure 6a compares the I–V curves with and without visible light illumination under zero gate bias voltage. A white light LED (Power ~36 mW) was used for illumination and the source-drain bias voltage was swept from -1V to 1V. In the dark, a linear I–V relation was measured with 0.048 Ω^−1^ conductance. Upon illumination, the device showed an increased current flow through the source-drain contacts with increased current flow at high bias voltage. The conductance of the graphene improved to 0.06 Ω^−1^, indicating a visible light photoresponse in our heterostructure. The calculated photo-responsivity of the heterostructure is about 278 mAW^−1^, which is much higher than that reported for modified graphene photo-detectors such as graphene metal junctions (6.1 mAW^−1^)^26^, graphene p-n junctions (10 mAW^−1^)^27^ and biased graphene (0.2 mAW^−1^)^28^. Note that our measurements were performed with a low intensity white LED light while modified graphene photo-response experiments were conducted under illumination from a high intensity laser source. The gate response under gate bias with and without light illumination is shown in Fig. 6b. In the dark, the transfer characteristic exhibited typical ambipolar behavior of graphene. The CNP was ~ + 1.5 V, indicating hole doping of the graphene layer. Under illumination, however, the response changed entirely. The source-drain current increased continuously as the gate voltage was varied from −20 V to 10 V. Multiple devices tested under the same conditions showed similar behavior. At positive gate voltage, the photoconductivity becomes much higher than that of the negative gate voltage region because photon induced electrons increase the net charge flow through the graphene. In the negative gate voltage region the induced holes reduce the net current between the source and drain. The output characteristics under illumination as shown in Fig. 6c clearly show the increase in photo-induced conductivity. While the gate voltage changed from −20 V to 20 V, the drain current also increased, corresponding to the increase of photo-conductivity. At V_g_ = −20 V, the graphene photoconductivity resulted in a conductance of 0.052 Ω^−1^ while it increased to 0.06 Ω^−1^ at 20 V. The difference between negative and positive gate voltage might be related to variations of net current flow through the channel. The response to turning the light on and off was measured under 0.2 V bias between source-drain as shown in Fig. 6d. The gate voltage was set to zero volts. Upon turning the light on the photocurrent sharply increased. When the light was turned off, the photocurrent quickly dropped to the dark state. The rise and fall times of our device were about 0.4 s. The fast temporal response is quite different from that of bare graphene and graphene hybrid photo-detectors that have a slow response to external light illumination with typical rise and fall times of several seconds^29^. Since the photo-excited electron-hole pairs in graphene recombine very rapidly due to the symmetrical electrode structure, the low light absorption (2.3% per mono-layer) of graphene, and the zero bandgap, the photo-response of graphene is weak. To improve its response, modified graphene structures such as graphene-metal junctions, graphene p-n junctions, graphene coupled to waveguides, and biased graphene have been proposed but the reported photo-responsivity is still in the range of 10 mAW^−1^, which is lower than that of our photo detector. Since the large bandgap (3.2 eV) of TiO_2_ does not allow for a photo-response to visible light, the photo-response observed here might originate from the Gr/TiO_2_ heterostructure. A possible mechanism for the enhanced visible light photoresponse seen in our Gr/TiO_2_ heterostructures may be provided by Geng, Liu and Yao^30^. In an attempt to explain the reported visible light photocatalytic effect of TiO_2_/graphene composites they calculated the electronic properties of atomic scale TiO_2_ clusters on graphene films and predicted anchoring of the clusters by charge rearrangement at the interface due to an interaction between the Ti 3d orbitals of the TiO_2_ and the p orbital of the graphene sheet. This results in extra states in the bandgap of TiO_2_ which enable absorption of visible light by the composite and the transfer of photoexcited electrons from the TiO_2_ valence band to the graphene conduction band. The subsequent separation of electrons and holes in graphene and TiO_2_ leads to long carrier lifetimes. In addition, since the interface between TiO_2_ and graphene plays a critical role, they also suggested that increasing the interface contact area could improve the photo response. We speculate that a similar process may be taking place in our comparatively large area heterostructures. The absorption of light takes place primarily in the TiO_2_ closest to the interface where their density of states is modified by the presence of the graphene layer. Electrons then transfer to the graphene layer and increase the electron density. The separation of electrons and holes reduces recombination and the holes in the TiO_2_ can screen the gating effect under positive bias while inducing more holes in the graphene layer under negative bias, leading to a reduced photoconductivity at negative bias. This model explains our experimental results shown in the gate response characteristics (Fig. 6b). In the modified graphene photo detector structures, the temporal light on/off photo-response showed slow rise and fall times because the photo-excitation is not related to a bandgap transition^31^. Therefore, we believe the abrupt on/off response seen in Fig. 6d is likely due to transitions between the TiO_2_ O 2p valence band and the graphene C 2p conduction band minimum of the Gr/TiO_2_ heterostructure.

## Conclusion

We demonstrated that C_60_ deposition produced graphene layers on transional metal layers (Ti) with a metal carbide interface layer (TiC). After O_2_ intercalation the byproduct TiC layer was completely converted into TiO_2_, which resulted in a graphene/oxide heterostructure. We showed that this heterostructure exhibits dual functional properties, showing both electronic field effect phenomena and an enhanced visible light photosensitity. The field effect phenomena and enhanced photosensitivity were demonstrated in bottom gated GFET devices.

## Methods

### Graphene growth

Metallic titanium layers (20 nm) were grown on an n-type SiC wafers by e-beam evaporation. C_60_ molecules were deposited on the Ti layers in a high temperature molecular beam epitaxy system. The growth temperature and carbon flux were 1400 °C and 1.6 × 10^−7^ Torr. The growth time was 30 min. For the study of graphene growth on TiO_2_ surfaces, the as-deposited Ti/n-SiC sample was annealed in an O_2_ environment at 800 °C for 1 hr. The oxidation of the metallic Ti layer was confirmed by XPS analysis. The graphene growth was performed with the same growth conditions as those for Ti/n-SiC. After graphene growth, the sample was exposed to air while being transferred to the XPS chamber for O_2_ intercalation experiments.

For the field effect transistor device tests, Au/Ti metal contacts (source and drain) were deposited on the graphene surface. The photo response experiment was performed by illumination with a white LED at room temperature.

### Calculation of activation energy

The temporal change in atomic concentration of TiC and TiO_2_ was estimated by high resolution XPS spectra. The rate constant (k) for TiO_2_ formation due to intercalation was obtained by analyzing the temporal change in the ratio of TiC to TiO_2_ atomic concentrations at each intercalation temperature. By fitting an Arrhenius plot (Log (k) vs. 1/T), the activation energy (E_g_) for oxidation was calculated. The graphene etching effect during O_2_ intercalation was monitored by the reduction of the graphene C 1s peak (284.7 eV) intensity. The etching activation energy was also calculated by estimating rate constant (k) and fitting the Arrhenius plot (Log (k) vs. 1/T). The temporal variation of C 1s ratio between as-grown and intercalated samples was used to calculate the rate constant (k) at each intercalation temperature.

### O_2_ Intercalation Procedure

After graphene growth, the sample was transferred to the load-lock of the XPS system. When the background pressure of the load lock chamber reached 3.0 × 10^−8^ Torr the sample was transferred to the analysis chamber. After initial XPS scans were acquired the sample was transferred back into the load-lock chamber for O_2_ intercalation. For the intercalation experiments research grade O_2_ was introduced into the chamber with a leak valve. The background pressure in the chamber was maintained at 1.0 × 10^−4^ Torr. Once the desired pressure was reached the sample was heated from the backside of the sample holder with a 10.6 μm CO_2_ IR laser. The laser output power was adjusted until the target temperature was reached. The temperature of the sample was measured with a pyrometer. After the intercalation the chamber was pumped down until a background pressure 3.0 × 10^−8^ Torr was reached. The sample was then transferred back into the analysis chamber for XPS data acquisiton.

The XPS data was acquired with an Scienta Omicron standard Mg Kα (1253.6 eV) X-ray source and Argus hemispherical analyzer. High resolution scans of the C 1s, O 1s, and Ti 2p regions were acquired at 20 eV pass energy with 0.1 eV steps. The binding energy scale was calibrated against the Au 4f_7/2_ (84.0 eV) peak with an overall resolution of ~1 eV. Each spectrum was fit with a Shirley background followed by fitting convoluted Gaussian and Lorentzian peak shapes for each chemical state in the region of interest. For the C 1s graphene and Ti 2p metal peaks an additional asymetry parameter was added to the Gaussian-Lorentzian convolution. The fitting procedure consisted of a Levenberg-Marquardt routine that minimizes χ^2^.

## Additional Information

**How to cite this article**: Park, J. *et al.* Approach to multifunctional device platform with epitaxial graphene on transition metal oxide. *Sci. Rep.* **5**, 14374; doi: 10.1038/srep14374 (2015).

## Supplementary Material

Supplementary Information
