# Supplementary material for: Approach to multifunctional device platform with epitaxial graphene on transition metal oxide
Source: Sci Rep. 2015 Sep 23;5:14374. doi: 10.1038/srep14374 (PMC4585821; doi:10.1038/srep14374)
Supplement: Supplementary Information [file srep14374-s1.pdf]

Supplementary Information

Approach to multifunctional device platform with epitaxial graphene on transition metal oxide

Jeongho Park[1]*, Tyson Back[1,2,3], William C. Mitchel[1], Steve S. Kim[1], Said Elhamri[4], John Boeckl[1], Steven B. Fairchild[1], Rajesh Naik[1], Andrey A. Voevodin[1]

[1] Air Force Research Laboratory, Materials and Manufacturing Directorate (AFRL/RXA)
Wright-Patterson AFB, OH 45433-7707

[2] University of Dayton Research Institute, , Dayton, Ohio 45469-0170, USA

[3] Center of Excellence for Thin Film Research and Surface Engineering, University of Dayton, Dayton, Ohio 45469-0170, USA

[4] Departments of Physics, University of Dayton, Dayton, Ohio 45469

* To whom correspondence should be addressed; electronic mail: jeongho.park@wpafb.af.mil

1. **Fitting Results for XPS of Ti 2p region**

   Figure 1 shows the fitting the results of Ti for the intercalation at 500 C. The bottom spectrum represents TiC. As the intercalation proceeds oxide formation, Ti $^{3+}$ and Ti $^{4+}$, can clearly be seen. After 720 minutes the sample was almost entirely oxidized. Intercalation experiments at higher temperatures proceeded in the same manner in a shorter period of time (see main text), 480 and 120 minutes for 600 and 700 C respectively.

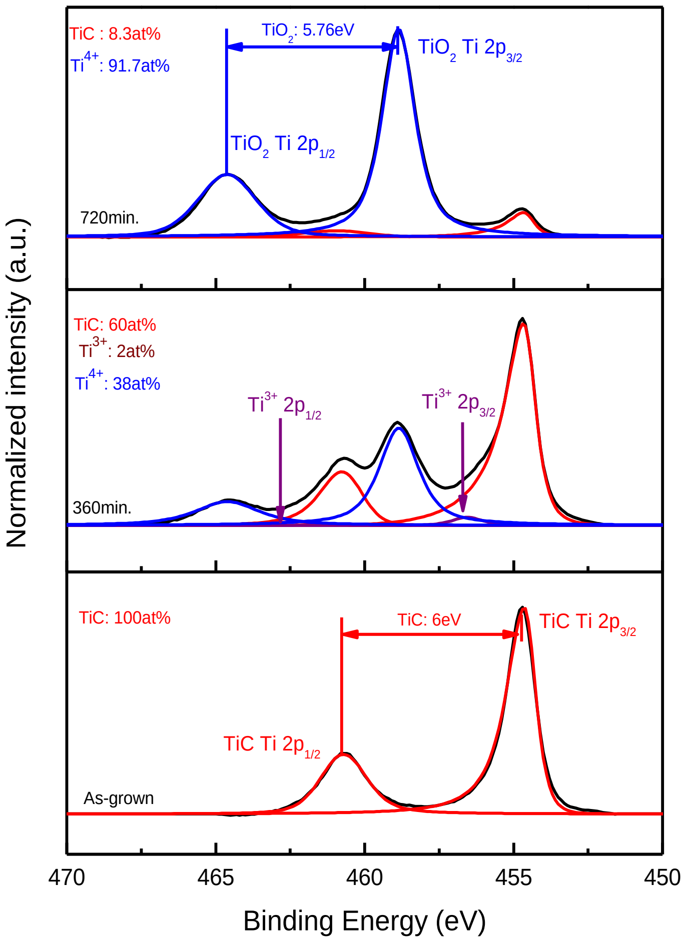

Figure S1 XPS of Ti 2p region for 500 ^{0}C intercalation. Spectra show the evolution of Ti as function of time with the bottom, middle, and top spectra representing 0, 360, and 720 minutes respectively.

2. **Leakage current measurement.**

The leakage current in the Graphene/TiO_2/n-SiC FET structure was measured. Au/Ti metal contact was deposited on top of graphene surface. For bottom contact, Ta metal was deposited on the back side of n-SiC substrate. The room temperature measurement was conducted in Air. The pA order of leak current was measured. During the transfer and FET characteristic measurement, the leak current was monitored. The measured current is in the range of pA order.

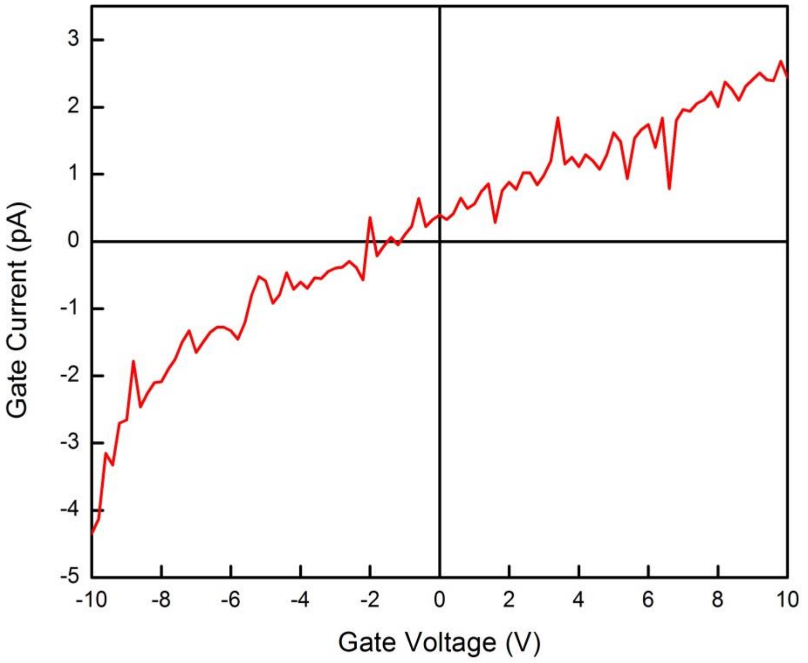

Figure S2. The leakage current measurement of Graphene/TiO_2 heterosturcutre

3. Raman analysis of graphene on Ti metal surface by C_{60} direct deposition

The uniformity of grown growth was estimated by large area Raman map. The Raman map was taken over 5mm x 5mm range with 300µm step to check uniform growth of graphene. The sample was grown with $1.6x10^{-7}$ torr C_{60} flux for 45min. at 1400°C. The 2D peak position was formed at 2725 ± 6cm^{-1}. As shown in Fig.S3a, the narrow variation of 2D peak position observed suggests that direct deposition of solid carbon source can provide uniform graphene layer. In addition, the quality of graphene was estimated by measuring the ratio of D band to G band in Raman spectra. The lower D/G ratio indicates the less defect density in grown graphene. As shown in Fig. S2b, we observed the D/G ratio showed inversely proportional to with growth time.

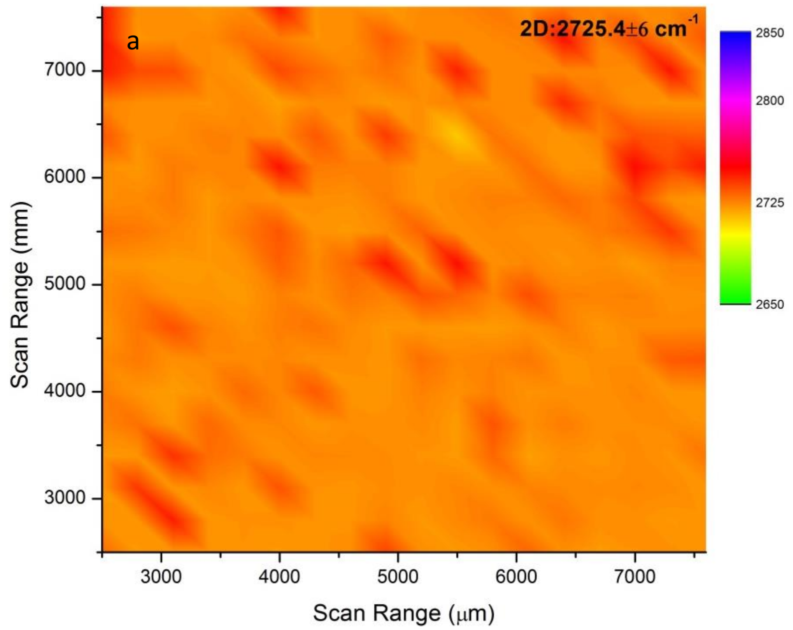

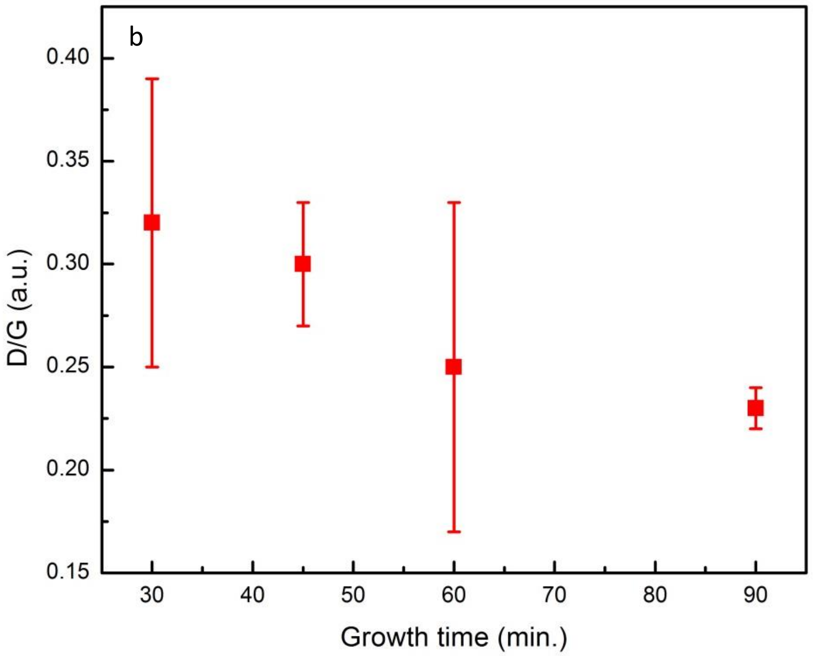

Figure S3. a. Raman map data of graphene grown on Ti metal substrate. B. D/G ratio variation with growth time.
